# Diagnosis and Surgical Treatment of Epilepsy

**DOI:** 10.3390/brainsci8070115

**Published:** 2018-06-21

**Authors:** Warren Boling

**Affiliations:** MD, FAANS, FRCSC, FRACS, Loma Linda University Medical Center, Loma Linda, CA 92354, USA; wboling@LLU.edu; Tel.: +1-909-558-4419

Epilepsy is a common neurological disease that can affect all ages. Although the majority of people with epilepsy will have excellent seizure control with medication, about 30% will fail anti-epileptic drugs. For those with medically intractable epilepsy, the recurrent seizures lead to increased mortality, risks of injury, and the seizures themselves are socially disabling [1,2,3]. Fortunately for many people with intractable epilepsy, epilepsy can be cured, or seizures better controlled with surgical treatment [4,5].

Localization of the seizure focus followed by surgical resection provides the best opportunity to cure the epilepsy, and a better understanding of the neuro-anatomy and physiology of epilepsy improves our ability to define the epileptic network and effectively treat the epilepsy [6]. An important advance that has improved patient care is minimal access surgical approaches, which result in more rapid recovery from surgery, less pain, and more satisfied patients [7].

Additionally, for individuals without an opportunity for cure of their epilepsy, new and emerging technologies have promise to reduce seizure frequency and severity, thus improving quality of life and preventing injuries and mortality that result from intractable epilepsy.

In this special issue, Boling et al. examines the profound negative consequences of medically intractable epilepsy that impacts the majority of the world’s population who reside in the developing world of Asia and sub-Saharan Africa. Stigma is a major driver of the significantly reduced quality of life of people with epilepsy, which is amplified in severely underserved and low-resource regions of the world due to poverty, severe treatment gaps, high mortality and morbidity of intractable epilepsy, lack of education and knowledge about epilepsy, and widespread misconceptions that epilepsy is related to witchcraft or sorcery as well as beliefs that epilepsy is contagious. Boling et al. then describe proof of principle in the developing world that surgery of medically intractable epilepsy can elevate quality of life and significantly reduce stigma.

Even in the developed world, due to seemingly inextricable reasons, wait times for patients with medically intractable epilepsy to be evaluated in a comprehensive epilepsy program are unnecessarily long. The delay to evaluation of surgically remedial epilepsies results in many patients being exposed far too long to the elevated mortality risk and reduced quality of life that results from intractable seizures. Sadanand explores this knotty problem using a novel mathematical approach of non-cooperative game theory. He then contrasts and compares the medical communities approach to glioblastoma multiforme, which has better defined treatment algorithms and expectations of care, with the medical community’s approach to intractable epilepsy treatment, and explains the discrepancies identified using game theory models. 

Anyanwu et al. reviews the definition of medical intractability and provides a broad overview of the treatment options available to patients who have failed medication. Although approximately 20 anticonvulsant medications are available in North America and Europe today, the authors point out that only two anticonvulsant medications need to be adequately trialed and failed prior to a patient being deemed intractable. The presurgical evaluation particularly EEG and imaging are discussed. Finally the various surgical procedures for both palliative and curative goals are covered.

New treatment options have recently become available for epilepsy patients that directly stimulate the brain to suppress the seizure focus. The Neuropace RNS system (Mountain View, CA, USA) is a closed loop device that monitors EEG in order to identify a seizure onset then delivers a stimulus to prevent the seizure from spreading to become symptomatic. Most recently, the FDA in the United States approved anterior nucleus of the thalamus stimulation for adults with medically intractable partial epilepsy using a Medtronic DBS stimulation device (Minneapolis, MN, USA). Two articles, one from Kwon et al. and another from Eastin et al. explore the recent developments and historical underpinnings of neuromodulation treatments of epilepsy. Eastin et al. reviews the various stimulation targets that have been explored for neuromodulation of epilepsy, and they discuss many of the individuals who have championed these efforts. Kwon et al. discuss mechanisms of action in neuromodulation treatment of epilepsy then the authors specifically explore randomized controlled trials of stimulation treatment strategies for epilepsy that have been published in the literature. The pioneering work of Irving Cooper in the 1970s was the first human brain stimulation performed for epilepsy, but randomized studies that followed soon after did not show benefit with a cerebellar target. Most of the modern interest in brain neuromodulation has focused on thalamic and supratentorial cortical targets, plus there is promising research targeting the hippocampus for stimulation. However, the most common neuromodulation strategy for epilepsy today continues to be vagus nerve stimulation. 

In this special issue, Boling describes the various surgical approaches, nuances and pitfalls of surgery of medically intractable temporal lobe epilepsy with an emphasis on mesial temporal lobe epilepsy (MTLE). Selective and keyhole approaches to treat intractable MTLE allow for more rapid recovery of the patient. Recently after two new laser ablation devices became available there has been a resurgent interest in thermal ablation as a treatment option for MTLE. Despite the advances of minimal access keyhole surgery and thermal ablation techniques, an accurate diagnosis of MTLE remains paramount in the surgical treatment success of medically intractable MTLE.

High quality imaging is a critical component of the evaluation of intractable epilepsy. Identification of a lesion aides in defining the epileptogenic zone and significantly improves the seizure freedom opportunity of surgery. Skull base temporal lobe encephaloceles are fascinating and often overlooked lesions that can result in temporal lobe epilepsy. Bannout et al. examined their groups experience with these lesions and reviewed the literature in order to determine if a limited lesionectomy of the encephalocele was sufficient or a more extensive anterior temporal lobe resection was required to achieve seizure freedom in cases of medical intractability. 

Finally, Hussein et al. evaluates in an animal model a common nutritional supplement l-Carnitine that seems to have an anticonvulsant effect. The authors identify that the current antiepileptic medications are ineffective in about 30% of people with epilepsy who will be intractable, and anticonvulsants used today mostly reduce neuronal excitation in order to lower the seizure threshold. In a rat model of epilepsy, the authors identified that l-Carnitine was associated with a marked reduction of the seizure frequency and shortened the seizure duration. Also, rats treated with l-Carnitine had relative neuroprotection with less neuronal death identified in the hippocampus. The beneficial effects of l-Carnitine were demonstrated by Hussein et al. to work through a novel mechanism of reduction of oxidative stress and up regulation of heat shock proteins. The authors caution that further research needs to be done to further elucidate mechanisms of action. However, l-Carnitine is promising as a novel class of anticonvulsant medication with a mechanism of action different from the standard anticonvulsants that lower the threshold of neuronal excitability.
